# Supplementary figures and images for: The genetic trail of the invasive mosquito species Aedes koreicus from the east to the west of Northern Italy
Source: PLoS Negl Trop Dis. 2025 Mar 31;19(3):e0012945. doi: 10.1371/journal.pntd.0012945 (PMC12005524; doi:10.1371/journal.pntd.0012945)

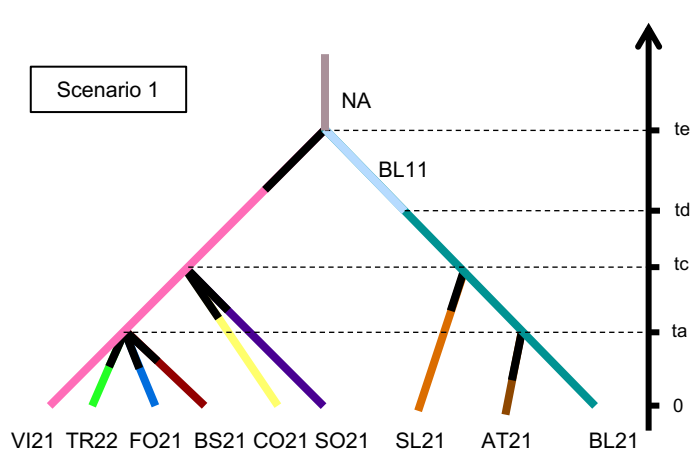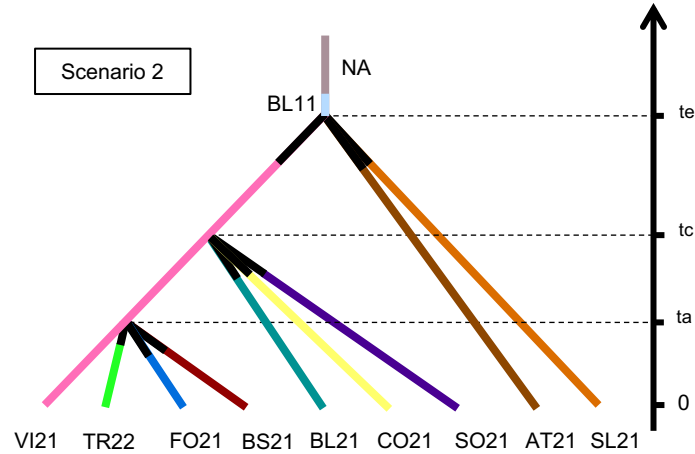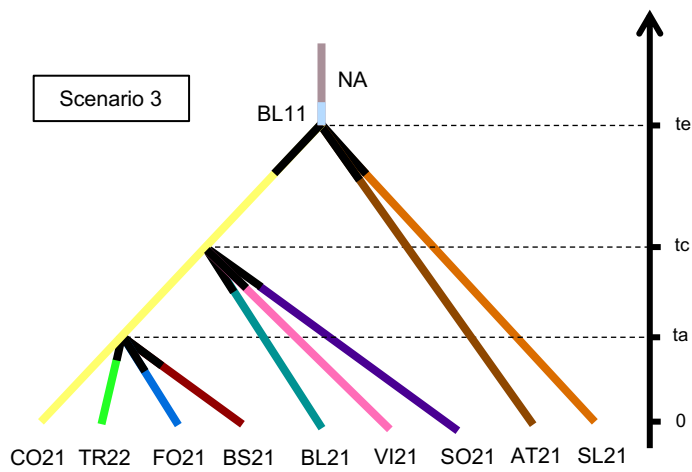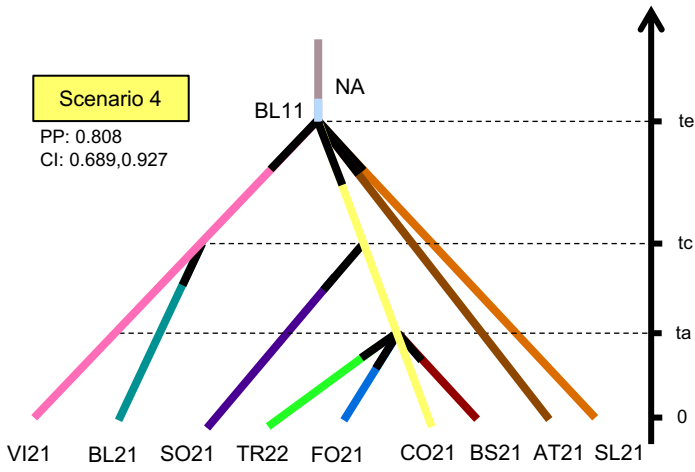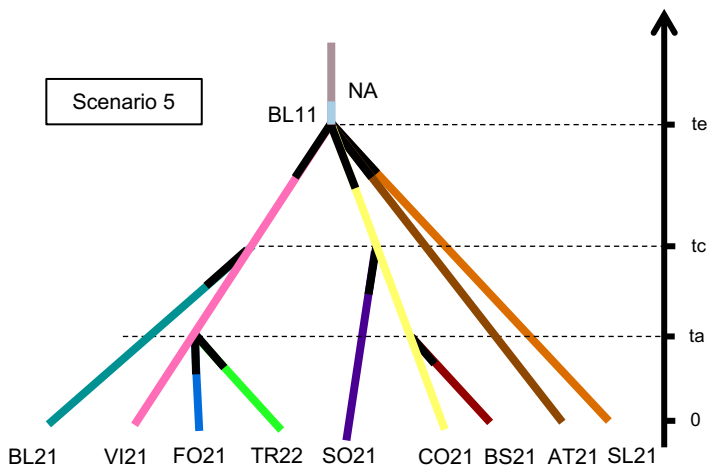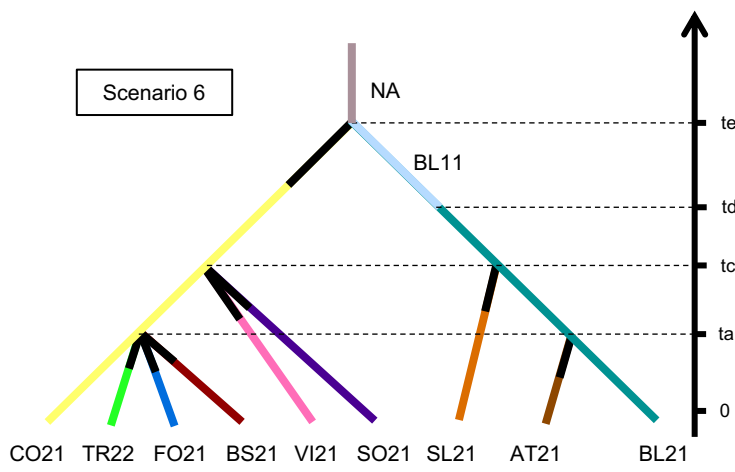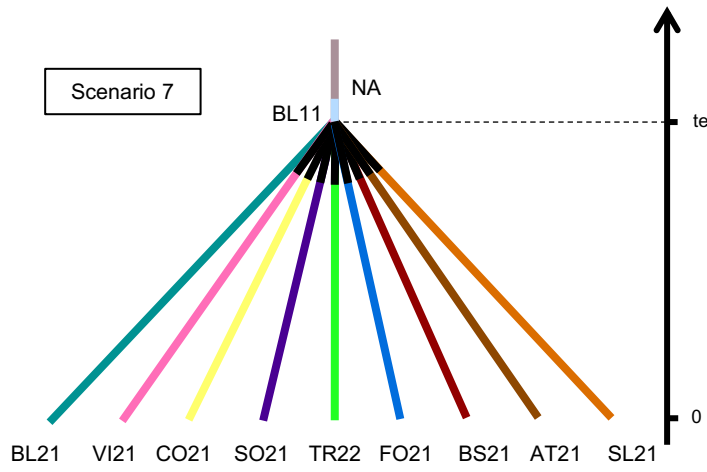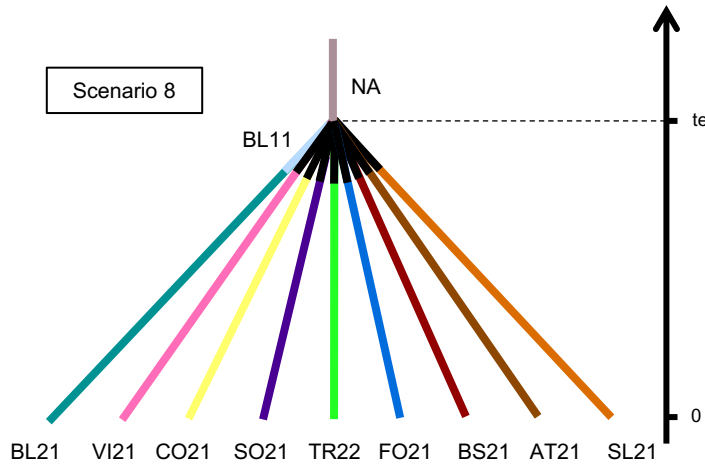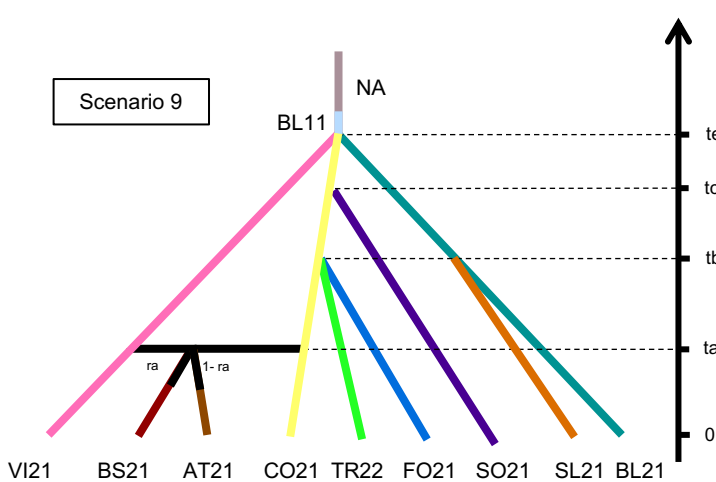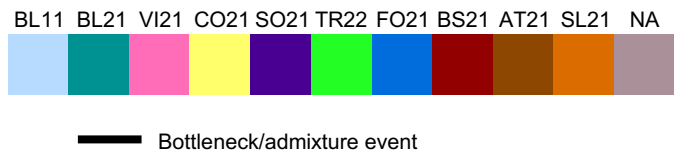

Supplement: S1 Fig — Representation of the scenarios designed and tested in DIYABC are reported. The scenario resulting as most representative (Scenario 4) on the basis of Bayesian computation is highlighted in yellow; for the latter, the posterior probability (PP) and confidence interval (CI) are reported. (PDF) [file pntd.0012945.s002.pdf]

GGD

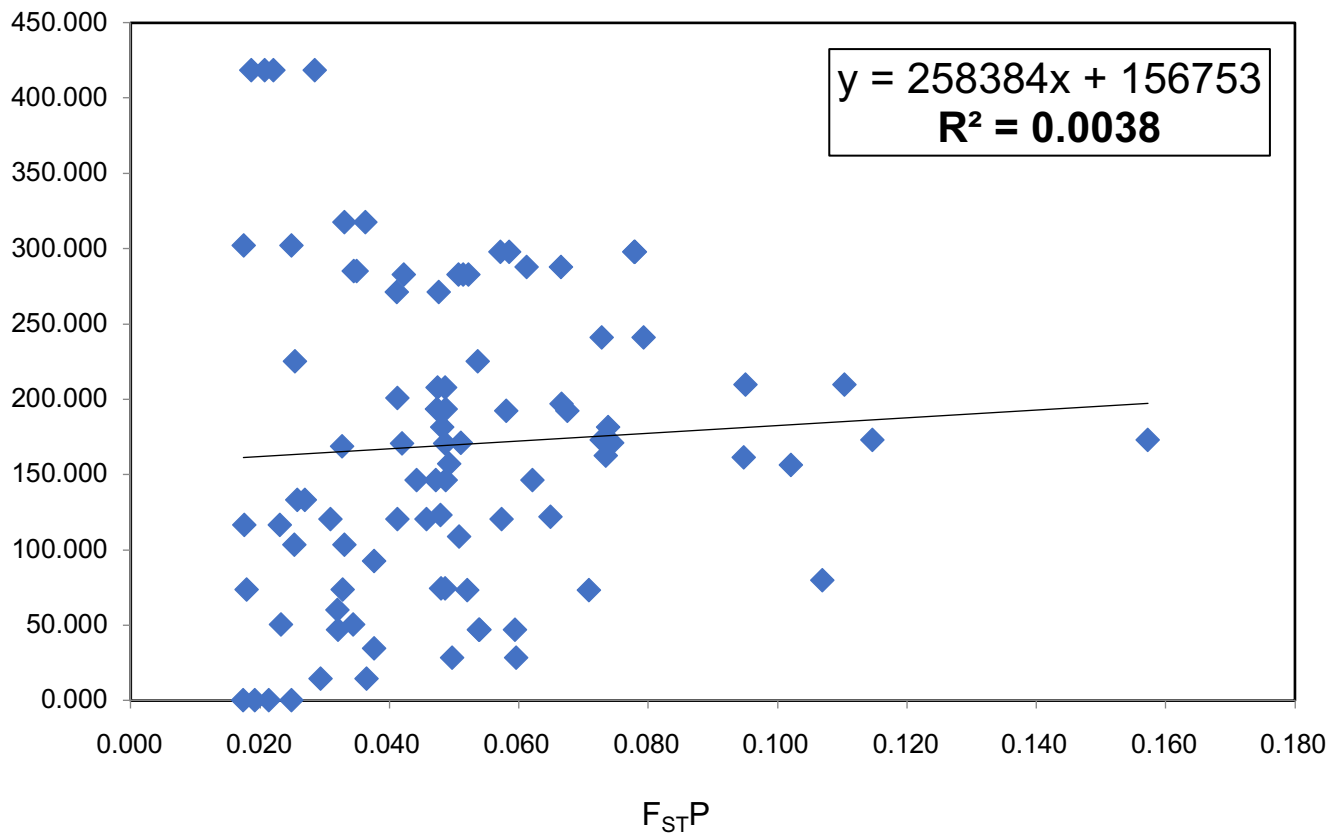

Supplement: S2 Fig — This is the result of 1000 permutations, performed to assess the possible isolation by distance in Ae. koreicus populations in Italy and Slovenia. (PDF) [file pntd.0012945.s003.pdf]
