# Supplementary material for: The genetic trail of the invasive mosquito species Aedes koreicus from the east to the west of Northern Italy
Source: PLoS Negl Trop Dis. 2025 Mar 31;19(3):e0012945. doi: 10.1371/journal.pntd.0012945 (PMC12005524; doi:10.1371/journal.pntd.0012945)
Supplement: S6 Table — The latter was produced as output of STRUCTURE HARVESTER, where are reported for each of the K tested: the number of repetitions (Reps), the mean value of the likelihood distribution (Mean LnP(K)) and the relative Standard Error, the mean of first rate of change of the likelihood K distribution (Ln’(K)), the mean absolute value of the 2nd order rate of change of the likelihood K distribution (|Ln’‘(K)|), the probability of K distribution obtained as: mean(|L“(K)])/ sd(L(K)) (ΔK). The two most probable co-ancestry distribution (K = 2, K = 3) are highlighted in bold. (PDF) [file pntd.0012945.s009.pdf]

K	Reps	Mean LnP(K)	SE LnP(K)	Ln'(K)	|Ln''(K)|	ΔK
1	20	-2866.690	0.273	NA	NA	NA
2	20	-2583.295	0.143	283.395	215.145	**1502.638**
3	20	-2515.045	0.343	68.250	113.105	**330.183**
4	20	-2559.900	4.446	-44.855	45.595	10.256
5	20	-2559.160	5.328	0.740	41.770	7.840
6	20	-2600.190	10.407	-41.030	24.590	2.363
7	20	-2665.810	9.312	-65.620	39.490	4.240
8	20	-2770.920	34.436	-105.110	2.795	0.081
9	20	-2878.825	33.723	-107.905	6.750	0.200
10	20	-2993.480	58.472	-114.655	68.310	1.169
11	20	-3039.825	51.963	-46.345	NA	NA
