# Supplementary material for: The genetic trail of the invasive mosquito species Aedes koreicus from the east to the west of Northern Italy
Source: PLoS Negl Trop Dis. 2025 Mar 31;19(3):e0012945. doi: 10.1371/journal.pntd.0012945 (PMC12005524; doi:10.1371/journal.pntd.0012945)
Supplement: S7 Table — The highest value is highlighted for each population of Ae. koreicus here analysed. (PDF) [file pntd.0012945.s010.pdf]

Population	K1	K2
BL11	0.015	**0.985**
BL21	0.009	**0.991**
VI21	0.120	**0.988**
CO21	0.005	**0.995**
SO21	0.005	**0.995**
TR22	0.109	**0.891**
FO21	0.006	**0.994**
BS21	0.010	**0.990**
AT21	0.008	**0.992**
SL21	0.011	**0.989**
KO21	**0.989**	0.011
