# Supplementary material for: The genetic trail of the invasive mosquito species Aedes koreicus from the east to the west of Northern Italy
Source: PLoS Negl Trop Dis. 2025 Mar 31;19(3):e0012945. doi: 10.1371/journal.pntd.0012945 (PMC12005524; doi:10.1371/journal.pntd.0012945)
Supplement: S8 Table — The highest value is highlighted for each population of Ae. koreicus here analysed. (PDF) [file pntd.0012945.s011.pdf]

Population	K1	K2	K3
BL11	**0.880**	0.015	0.105
BL21	**0.841**	0.009	0.160
VI21	**0.697**	0.013	0.290
CO21	0.145	0.006	**0.849**
SO21	0.135	0.006	**0.859**
TR22	0.314	0.092	**0.594**
FO21	0.095	0.007	**0.898**
BS21	0.419	0.018	**0.563**
AT21	**0.549**	0.010	0.441
SL21	**0.844**	0.010	0.146
KO21	0.012	**0.978**	0.010
