# Supplementary material for: The genetic trail of the invasive mosquito species Aedes koreicus from the east to the west of Northern Italy
Source: PLoS Negl Trop Dis. 2025 Mar 31;19(3):e0012945. doi: 10.1371/journal.pntd.0012945 (PMC12005524; doi:10.1371/journal.pntd.0012945)
Supplement: S9 Table — Highest values of posterior probability are highlighted, and resulted all associated to Scenario 4 in all the tested datasets (n). (PDF) [file pntd.0012945.s012.pdf]

Scenario	Posterior probability	Confidence Interval
1	0.006	0.000, 0.021
2	0.004	0.000, 0.002
3	0.030	0.004, 0.057
4	**0.808**	**0.689, 0.927**
5	0.002	0.000, 0.017
6	0.012	0.000, 0.026
7	0.003	0.000, 0.018
8	0.008	0.000, 0.022
9	0.128	0.025, 0.232
