# Supplementary material for: The genetic trail of the invasive mosquito species Aedes koreicus from the east to the west of Northern Italy
Source: PLoS Negl Trop Dis. 2025 Mar 31;19(3):e0012945. doi: 10.1371/journal.pntd.0012945 (PMC12005524; doi:10.1371/journal.pntd.0012945)
Supplement: Attachment — Submitted filename: Rebuttal_PnTD_PG.docx [file pntd.0012945.s015.docx]

**General Considerations.**

We would like to thank all the Reviewer for their comments and suggestions. We realized that there were some incongruencies between the statement of the aims and what we can describe from the analyses we carried out. Particularly, we cannot really discuss or have hints about the origin of the invasion of *Ae. koreicus* in Italy. We would like to be able to answer this question, but retrieving the samples from multiple locations from the native area turned out to be really difficult. Despite our efforts (more than 2 years) we were able to collect only one population, that was included in the analyses.

For this reason, we decided to focus the manuscript on the possible number of introduction events in Italy, the possible dispersal patterns and the analysis of the overwintering populations. We left the Korean sample in the analysis, briefly stating that the population we have analyzed is not the source of our invasive populations, to genetically support the morphological evidence of the existence of two distinct morphotypes in the species.

We think that the revised manuscript is better focused and does not overstate the results and does not create unfilled expectations.

**Reviewer 1**

- Methods

**A lot of data analysis were done but mostly not necessary to answer the research objectives.**

Re: The Reviewer has opened the discussion about a lot of interesting points, thank you very much. We will reply point by point aiming to clarify all the criticisms that were mentioned.

**I suggest to add additional samples from endemic countries such as China, Russia, other sites in Korea.**

Re: As stated, we would like to be able to perform these analyses, but the collection of these additional sample is out of our possibilities in a brief time period. We therefore decided to focus our Discussion on the genetic relationship among the Italian and Slovenian samples.

**The information about the number of samples per population should also be mentioned in the methodology. Sample size is sufficient if the objectives is to know the population genetic structure of the Ae. kroeius.**

Re: The number of specimens considered for each population is specified in Table 1 in the Methodology section, in the column “N”. As kindly suggested, we have added a footnote hoping to make it clearer. In addition, the number of samples included in the population genetic analyses after the removal of siblings is specified in Table 3 in the Results section (also in this case, the number of individuals is reported in the column “N”).

**However, it was mentioned that the objectives "we aimed at elucidating the processes driving its invasion dynamics in Italy, and potentially in the rest of Europe.” So in this regard, I suggest to add samples from endemic countries and other neighboring European countries.**

Re: We have modified this sentence, and moved the focus on Northern Italy, which is the geographic region the genetic investigation is centered on. As for samples from the endemic area, it would be fundamental in future studies to include more individuals from other European countries to obtain more comprehensive data about the invasive dynamics of *Ae. koreicus* at continental level.

**I suggest to perform UPGMA dendrogram to determine genetic groups and also analyze the migration rate between the sites using Geneclass.**

Re: We think that the analyses done in the Manuscript, which were performed based on the previous expertise of the authors, are already addressing these questions and we preferred not to perform new analyses to avoid making the text too dense. Specifically, genetic distances and relationships characterizing populations of *Ae. koreicus* were deduced with the computation of F_ST_P values (which are the basis of UPGMA dendrograms). These were also highlighted in PCA and DAPC plots (Figs 2A and 2B in the new version of the Manuscript), which allowed the visualization of the relation among individuals and populations, and potentially the subsistence of genetic groups. Indeed, in the DAPC the clusterization of populations from Northern Italy is evident, and also the grouping of populations from the Central part of Northern Italy (i.e. CO21, SO21, SO22, TR22, FO21, FO22) vs the individuals from the Eastern part of the Italian country. This was then confirmed by the Bayesian computations of STRUCTURE, from which the genetic structuring of populations was deduced (Fig. 3 in the new version of the Manuscript). Last, we think that the subsistence of gene flow is evident in the PCA plot (Fig. 2A of the revised Manuscript), even considering high genetic homogeneity of the individuals we have analysed.

**Testing the isolation by distance using mantel test is also suggested. There are many unnecessary data analysis performed but it doesn't answered well your research objectives. I also suggest to determine if the genetic distance is affected by geographic distance so a spatial autocorrelation test is also suggested.**

Re: A Mantel test was performed, and the related graphs is reported in the Supplementary Information (Fig S2). The latter was performed comparing genetic (F_ST_P values) and geographic distances (a GGD matrix was computed using mean geographic coordinates associated to each population), and no correlation between the variables was observed. Based on this, genetic distances are not affected by the geographical origins of the samples.

- Results

**The results are not completely presented and most results are not necessarily main and i suggest to put it as supplementary file. please see further comments in the attached file.**

Re: A more detailed response can be found in the following sections.

- Conclusions

**The limitations of the study was mentioned however this limitation is necessary to answer the research objective so i suggest to add aditional samples or revise your research objectives.**

Re: We have amended the research objectives, as already mentioned.

- Summary and General Comments

**L54-55 Do you mean 14 populations in total, 13 from Italy and Slovenia and one from Korea? Please confirm and revise if necessary.**

Re: The authors have updated the sentence.

**Table 1. please put the meaning of abbreviation, N under the table.**

Re: N indicates the number of samples analysed for each population. This was reported in the in the caption of the Table, and we have now added it as footnote too.

**For table 4, I suggest to put it as supplementary file and make it more organized, it is too messy to look at.**

Re: We have modified the table as follows: F_ST_Ps values were kept in the main text (Table 4), while the 95% confidence intervals were moved in a separate Table which was added as part of the Supporting Information (S5 Table).

**In the methodology, I suggest to include a supplementary table of the list of the microsatellites and their reference journal.**

Re: We have added the microsatellites list, together with the doi reference of the publication as S2 Table.

**L562-567 are redundant and was mentioned already previously.**

Re: The Discussion section was revised, making it more smooth and less redundant.

**L581-583 I agree that including a population from the endemic region is important however samples were only collected from one site and I suggest to include representative samples from other sites and or other endemic countries so you can compare the genetic structure and relatedness of the samples from Italy.**

Re: As already stated, unfortunately, it was not possible to include more samples in the present study which, even for this reason, was focused on the pattern of expansion of *Ae. koreicus* in the Italian and Slovenian countries. Nevertheless, we believe that the genetic distance between the Korean population and the Italian and Slovenian ones reflects the visualization of their different morphotypes. For this reason, we kept the Korean population in the manuscript.

**L587-588 This is a strong claim that the samples are not related from ROK since you only sampled few individuals and from a single site which is can also be a factor.**

Re: The sentence was rephrased (Lines 560-561) to clarify that a genetic relation was not detected with that particular population from the ROK.

**I suggest to deposit your dataset in publicly available database for example** [**vectorbase.org**](http://vectorbase.org)

Re: Our dataset is already publicly available, and can be found in Zenodo at the following link: [doi.org/10.5281/zenodo.12800346](http://doi.org/10.5281/zenodo.12800346). We have specified now it in the Methodology part of the Manuscript (Lines 188-189), we hope this could make it more clear.

**The authors have done extensive data analysis however it seems that some data exploited are not necessarily needed to answer their objective to “population genetics of *Ae. koreicus*, with a particular focus on the populations dispersed throughout the pre-alpine area of Northern Italy. Through comprehensive population genetic analyses, we aimed at elucidating the processes driving its invasion dynamics in Italy, and potentially in the rest of Europe.” If these is the main objective it is necessary to sample individual mosquitoes from native countries of *Ae. koreicus* such as in China, Russia and from neighboring European countries. The results does not wholly answered the objectives however it showed preliminary data supporting the genetic structure of the mosquitoes in Italy, I suggest to revise the objectives and focus mainly on the population genetics of *Ae. koreicus* in Italy and remove the samples from Korea in all the data analysis.**

Re: We have corrected the senteces about the aim of the present Manuscript, to underly that the focus is the invasive situation in Northern Italy.

**Additional data analysis are needed to answer the research questions. The discussion part should be improved more by discussing more about how their study is novel and how it answer research gaps. The novelty and importance of their study was not highlighted.**

Re: We have revised the Discussion, and tried to make the novelty of the study more evident.

**Reviewer 2**

- Editorial and Data Presentation Modifications?

**Below, some minor comments to the paper:**

**- Line 141: it is clear from your table but I would mention 10 collection sites in this sentence instead of 14, and specify that for 4 collection sites, you include a temporal component to the investigation (which I highly appreciate).**

Re: We have modified the paragraph as suggested (Line 137 and Lines 141-143).

**- Line 157: could you specify if you visited the collection sites multiple times over year- and if so at which intervals**

Re: In Lines 151-154 we have now added more details about the number of collections and their time frame.

**- Line 221: I do not understand this sentence: could you please rephrase**

Re: Done (Line 208). We hope it is clearer now. **- Line 353: add a reference to the sentence**

Re: The reference was added; in addition, as suggested by Reviewer 1, the list of microsatellites and primer pairs for their amplification was reported in S2 Table.

**- Line 412 and 415: once indicated 10000, once 1000; a typo?**
Re: That was a typo. We corrected the valued in the legend of the Table. Notably, the 95% confidence intervals related to F_ST_P values have now been moved in S5 Table, to allow an easier visualization, as suggested by Reviewer 1.

**- Line 421-423: could you clarify this statement**

Re: The misleading statement was rephrased. **- Line 430: did you also check differences in rare alleles between populations collected at the same sites over multiple years?**

Re: The number of rare alleles was computed for all populations, and is reported in S4 Table (column “Np”). However, the fixation of rare allele was noticed in very few individuals over the years.

**- Line 530: referring to your first table, samples from tr22 were collected in 2022 and not 2021**

Re: We have corrected this misleading sentence (Lines 485-486).

- Conclusions

**Discussion: it would be worth including a paragraph about the methodological limitations as after all, the amount of SSR markers investigated in this study is quite limited.**

Re: The suggested paragraph has been added in the Discussion section (Lines 567-572).

- Summary and General Comments

**The present research employs recently described SSR markers to contribute unraveling the introduction pathways and invasive potential, as well as colonization pattern of *Aedes koreicus* in northern Italy and parts of Slovenia. I must admit that I have not much to comment as the paper is already well-developed, complete (including very clear figures), fluently written, with a clear and logical structuring. The methodology is thoroughly explained, and the paper contributes to the field of invasive species colonization patterns. I therefore recommend the paper for publication in PLOS Neglected Tropical Diseases, also since it fits the journal scope. Congratulations on this study.**

Re: The authors again profoundly thank the Reviewer for this judgment and for all the suggested corrections.

**Reviewer 3**

**The authors of this study investigate the genetic structure of a mosquito population and hypothesize its expansion potential. *Aedes koreicus*, whose adaptive capacity and vectorial potential remain poorly understood and highly concerning, is the subject of a detailed and well-articulated analysis. The manuscript is well-written, with fluent English and an in-depth investigation of genetic correlations. Studies like this, which aim to trace the introduction pathways of *Aedes koreicus* and understand its past and future dispersal patterns, are crucial for effective prevention and control measures against this mosquito and other potential invasive species. Anyway, some revisions are necessary.**

1. **In the Introduction, lines 104–111, the discussion of *Ae. koreicus* vectorial capacity worldwide includes studies up to 2022. Could this section be expanded? Are there no more recent studies, from the past two years, about this topic?**

Re: Unfortunately, there are no other published and more recent studies focused on the vector competence of *Ae. koreicus*. However, we can anticipate that Dr. Fabrizio Montarsi and Dr. Francesco Gradoni, both co-authors of the present article, are investigating the possible role of *Ae. koreicus* in the transmission of Dengue.

2. **The analyses clearly show that individuals from the Republic of Korea are not genetically related to the populations sampled in Italy and Slovenia. Before conducting the microsatellite analyses, was the species identity of the ROK population verified? Were the samples from this population, as well as those from Italy and Slovenia, previously sequenced for *Cytochrome oxidase I*?**

Re: Yes, we have amplified and sequenced the *Cytochrome oxidase I* (COI) region of few specimens using the LCO1490 and HCO2198 primers (Folmer et al., 1994; Arnoldi et al., 2022). Specifically, we have tested samples from Italy and from the Republic of Korea. Results of Sanger sequencing revealed that the analyzed mosquitoes were correctly identified as *Ae. koreicus*, with no differences in the sequence of the COI gene from the native and invasive area.

3. **The results, presented in numerous tables and figures, are comprehensive and well-explained, but their abundance makes the text somewhat challenging to read. I suggest combining Figures 2 and 3 into a single panel to streamline the discussion and improve readability.**

Re: The authors thank the Reviewer for the suggestion. We have now merged Figures 2 and 3 in a single panel, named Figure 2 in the new version of the Manuscript.

4. **A similar suggestion applies to the Discussion section, which feels dense. The introductory part, which overlaps with the Introduction section, could be shortened. I also suggest identifying other redundant sections that might be simplified, as they are already thoroughly addressed in the Results.**

Re: The Discussion section has been revised, according to the kind suggestion of the Reviewer. Now it should be less redundant and, hopefully, clearer to read.

5. **Why did the authors choose microsatellites as genetic markers for this study? While advantageous, due to their abundance and high mutation rate, microsatellites also have limitations, such as mutational biases and low standardization. Did the authors consider using more reliable and automatable techniques, such as SNP analysis?**

Re: Thanks to the reviewer for this comment, which is totally on point. Indeed, the analysis of SNPs results is more powerful and reliable conclusion in the field of population genomics, since more information can be recovered. We have added a comment about the power of resolution of microsatellites in the Discussion (Lines 567-572), even in relation to the quite low number of markers that we employed, as suggested also by Reviewer 2.

We choose to develop a panel of microsatellites and use them for the present analyses since poor genomic resources specific for *Ae. koreicus* were available when the study started. Nevertheless, beside no genomic assembly was available, it was possible to recover informative microsatellite-containing sequences bioinformatically (Teekema et al., 2022; Soresinetti et al., 2023, for the detailed methodology), resulting in reliable analyses of the genetic patterns of populations of *Ae. koreicus*. However, a reference genome for the species was recently published (Accession number: GCA_024533555.2; Nagy et al., 2024), and we are now proceeding with the whole-genome sequencing of various individuals of *Ae. koreicus* to recover more robust conclusion about the genetic history of this mosquito species.

**Specific comments on text formatting:**

- **Line 571: "population, or"**
- **Line 618: "population, different"**
- **Line 687: "structuring, revealed by STRUCTURE, seems"**

Re: The authors thank the Reviewer for the comments about the format of the text. All sentences have been modified during the revision of the Discussion.

Bibliography of the Rebuttal

Arnoldi I, Negri A, Soresinetti L, Brambilla M, Carraretto D, Montarsi F, et al. Assessing the distribution of invasive Asian mosquitoes in Northern Italy and modelling the potential spread of *Aedes koreicus* in Europe. Acta Trop. 2022 Aug;232:106536.

Folmer, O., Black, M., Hoeh, W., Lutz, R., Vrijenhoek, R., 1994. DNA primers for amplification of mitochondrial cytochrome c oxidase subunit I from diverse metazoan invertebrates. Mol. Mar. Biol. Biotechnol. 3, 294–9.

Nagy NA, Tóth GE, Kurucz K, Kemenesi G, Laczkó L. The updated genome of the Hungarian population of *Aedes koreicus*. Sci Rep. 2024;14(1):7545.

Soresinetti L, Arnoldi I, Negri A, Naro G, Michelutti A, Montarsi F, et al. Development of microsatellite markers for the invasive mosquito *Aedes koreicus*. Parasit Vectors. 2023;16:223.

Teekema, S., Stroo, A., Uiterwijk, M., van de Vossenberg, B., Jacobs, F., IbanezJusticia, A., 2022. First finding of *Aedes koreicus* (Diptera: Culicidae) in the Netherlands. JEMCA 1–8. https://doi.org/10.52004/JEMCA2021.0005.
