# Supplementary material for: The genetic trail of the invasive mosquito species Aedes koreicus from the east to the west of Northern Italy
Source: PLoS Negl Trop Dis. 2025 Mar 31;19(3):e0012945. doi: 10.1371/journal.pntd.0012945 (PMC12005524; doi:10.1371/journal.pntd.0012945)
Supplement: Attachment — Submitted filename: Review PLOS.docx [file pntd.0012945.s013.docx]

The authors of this study investigate the genetic structure of a mosquito population and hypothesize its expansion potential. *Aedes koreicus*, whose adaptive capacity and vectorial potential remain poorly understood and highly concerning, is the subject of a detailed and well-articulated analysis. The manuscript is well-written, with fluent English and an in-depth investigation of genetic correlations. Studies like this, which aim to trace the introduction pathways of *Aedes koreicus* and understand its past and future dispersal patterns, are crucial for effective prevention and control measures against this mosquito and other potential invasive species. Anyway, some revisions are necessary.

1. In the Introduction, lines 104–111, the discussion of *Ae. koreicus* vectorial capacity worldwide includes studies up to 2022. Could this section be expanded? Are there no more recent studies, from the past two years, about this topic?

2. The analyses clearly show that individuals from the Republic of Korea are not genetically related to the populations sampled in Italy and Slovenia. Before conducting the microsatellite analyses, was the species identity of the ROK population verified? Were the samples from this population, as well as those from Italy and Slovenia, previously sequenced for *Cytochrome oxidase I*?

3. The results, presented in numerous tables and figures, are comprehensive and well-explained, but their abundance makes the text somewhat challenging to read. I suggest combining Figures 2 and 3 into a single panel to streamline the discussion and improve readability.

4. A similar suggestion applies to the Discussion section, which feels dense. The introductory part, which overlaps with the Introduction section, could be shortened. I also suggest identifying other redundant sections that might be simplified, as they are already thoroughly addressed in the Results.

5. Why did the authors choose microsatellites as genetic markers for this study? While advantageous, due to their abundance and high mutation rate, microsatellites also have limitations, such as mutational biases and low standardization. Did the authors consider using more reliable and automatable techniques, such as SNP analysis?

**Specific comments on text formatting:**

- Line 571: "population, or"
- Line 618: "population, different"
- Line 687: "structuring, revealed by STRUCTURE, seems"
